# Rejected Cases

**Published:** 1884-09

**Authors:** E. W. Berridge

**Affiliations:** London


### CLINICAL BUREAU.

---

### REJECTED CASES.

E. W. Berridge, M. D., London.

The following cases were sent to the physician (a *professed* homœopath) who is compiling *Kali bichrom.* for the new edition of the *Materia Medica* of the Hahnemann Publishing Society. They were rejected because they were cured with the Cm potency! Perhaps they ought not to have been cured; or, being cured, ought to be concealed from the public eye, lest the mongrels should be asked awkward questions as to their repudiation of the highest potencies. Surely, to slightly alter the words of the poet:

"The force of *bigotry* could no farther go."

Case I.—Miss —— had for two days pain in left malar *bone*, worse on coughing, with soreness of the *bone* to touch. At 3 P. M. I gave one dose of *Kali bichromicum*$^{cm}$ (Fincke). She improved soon after the dose, and by 9 P. M. was quite well.

Case II.—February 9th, 1871. Miss —— æt. nineteen. Has been ill for a week; shooting inward in left malar *bone*, with pressure, and for three days also the same pains across bridge of nose. Feels hot and cold alternately. The pain makes her feel inclined to cry. Sometimes shooting in the *bone* over left eye, cough worse in morning; it hurts the painful part of cheek; sputa, greenish-yellow, bitter. Gave one dose of *Kali bichrom.*$^{cm}$ (Fincke).

February 16th. Reports that the neuralgia went the same day. The chills and heats went on 11th. Cough better; no expectoration since 13th. Soon became quite well.

These two cases verify the action of *Kali bichrom.* on the *bones* of the face, as recorded in the provings. It is the only remedy which has "pains in *bones* of face on coughing" (see Lee & Clark's *Cough Repertory*, p. 44). In the second case, the facial pains proceeded from left to right. In the provings of Zlataravitch they proceeded from right to left. This is another verification of Hering's law of *Inverse Directions*, which is really an extension of or corollary to HAHNEMANN'S doctrine of the nature of chronic diseases and their eradicative treatment.

CASE III.—October 19th, 1881. Mrs. W——. Heartburn on first lying down at night; worse by lying on right side; sometimes she falls asleep without it; but then wakes with it; it sometimes occurs after meals; aggravated by salt meat or salt fish; has suffered thus for a long time, and has been worse for the last month or two. Gave *Kali bichrom.*$^{cm}$ (Fincke) every morning for seven days.

October 28th. Reports that the heartburn quite ceased after the first dose; it is not now excited by lying on right side, though it still feels as if it would come on; has eaten salt meat with impunity.

November 26th. No return of heartburn. No further report.

CASE IV.—Mr. F. V., æt. eighteen. March 19th, 1881. Has been ill since 1877 with the following symptoms: Mucus from throat, sometimes black, brownish, or yellow, generally sinking in water. Yellow nasal discharge, smelling badly, especially when he has a cold. Very often hoarse, with dryness of throat in evening. Nose generally most stuffy in morning. When the nose is especially stopped up, there is headache. Throat and mouth parched on waking and nose stopped up. Has used Tannin locally, and all sorts of sprays, paints, and gargles, but without relief. Gave *Kali bichrom.*$^{cm}$ (Fincke) every morning for seven days.

March 27th. Reports decided improvement, but still has the obstruction in nose when speaking.

April 11th. Continued improvement; very much better, notwithstanding a cold caught at the boat-race which caused an "awfully sore throat."

April 27th. Still improving, though there remains considerable discharge from nose; discharge from nose and throat has lost its bad smell and dirty color. Mucus from throat often quite black. Sleeps much better, and wakes in morning with throat and mouth moist and nose clear, allowing easy breathing. Catarrh quite gone. Feels very much better generally; stronger, more vigorous.

May 4th. Still improving, though not so markedly or so rapidly as at first, but is better in every way; stronger, more life in him. A few small pimples on face.

May 24th. Saw him for the first time, having treated him hitherto by correspondence. He reports still further improvement, though still at times troubled with attacks of stuffiness, which he says are becoming "like angels' visits, few and far between." No further report.

Why were these cases rejected? Echo answers, Why?

